# Microtia Ear Reconstruction Using Tissue Expander and Autologous Costal Cartilage: Our Experience and Comparing Two Age Groups

**DOI:** 10.29252/wjps.8.3.324

**Published:** 2019-09

**Authors:** Sanjib Tripathee, Meng Xiong, Jue Zhang

**Affiliations:** Zhongda Hospital, Southeast University, Nanjing, China

**Keywords:** Microtia, Ear, Reconstruction, Tissue expander, Costal cartilage

## Abstract

**BACKGROUND:**

Ear reconstruction is one of the most challenging surgeries faced by reconstructive surgeons because of its complex three-dimensional structure. Various surgical methods and materials have been used over the years. The process of microtia reconstruction using tissue expander is performed in three stages of first that is implantation of tissue expander, second stage involves framework fabrication using autologous costal cartilage and implantation in the pocket and third stage involves tragus and concha reconstruction.

**METHODS:**

Totally 180 cases of microtia reconstruction using tissue expander and autologous costal cartilage over 2 years were enrolled, while two age groups were compared regarding operative time, tissue expansion, number of autologous costal cartilage harvested and complications during and after reconstruction.

**RESULTS:**

The overall complication in microtia reconstruction was 25%. No major difference was found between complication rates among the 2 age groups. Similarly, no significant difference was found between two groups in term of surgical time and tissue expansion. The major difference was found in number of costal cartilage harvested for the framework fabrication among the two groups.

**CONCLUSION:**

Microtia reconstruction using tissue expander and autologous costal cartilage is a standard method of ear reconstruction with good satisfaction rate for surgeons and patients. Although the complication rate was high in our study, most of the cases were managed with acceptable results. Therefore, a standard protocol should be developed regarding the timing of the surgery for microtia reconstruction, considering pre-operative radiological analysis of the costal cartilage development along with age and weight of the patient.

## INTRODUCTION

Microtia is a congenital malformation of the external ear that ranges in severity from mild structural abnormalities to complete absence of the ear (anotia). The external ear is a critical component for overall aesthetic balance of the face, and slight deformity of the ear is easily visible. Ear reconstruction is one of the most challenging surgery faced by reconstructive surgeons because of its complex three-dimensional topography and need to construct near normal external ear.^1^^,^^2^

Various techniques and materials have been used over the years to reconstruct normal looking and durable external ear. The modern era of auricular reconstruction began with Tanzer who reintroduced the technique of autologous costal cartilage grafts as a method of auricular reconstruction.^3^ A significant majority of surgeons worldwide continue to use techniques using autologous rib cartilage to reconstruct the auricular framework. According to the national survey of American Society of Plastic Surgeons, 91.3% of the plastic surgeons choose autologous cartilage staged reconstruction for microtia reconstruction.^4^

The technique described by Brent, Nagata and Firmin are most widely used techniques for the microtia reconstruction.^2^^,^^5^^,^^6^ Various modifications have been made to these techniques over the years. In our institution, we used modified Nagata technique for the ear reconstruction using tissue expander. Although there is no universal consensus regarding the timing of microtia surgery, most reconstructive surgeon perform microtia reconstruction after the patient is 6 years old in China.^7^

Some surgeons argue that reconstructive surgery for microtia should be delayed till the child is 10 years old to reduce the complication associated with the chest deformity. The complication rate is significantly high in chest donor site if the cartilage is harvested at the age smaller than 10 years old.^7^ Our study would focus on complications associated with microtia reconstruction in the recipient site and further compared two age groups in terms of surgical time, tissue expansion, number of autologous costal cartilage harvested and complications after reconstruction.

## MATERIALS AND METHODS

We perform microtia reconstruction in our center based on modified Nagata technique. Nagata advocated that first surgery should not be performed earlier than 10 years of age or chest circumference at level of xiphoid grows to at least 60 cm. We have been performing microtia surgery as early as patient reach 4 years old. Therefore, we divided patients into 2 groups to evaluate significant different on cartilage harvest, surgery and complication rate. The process of microtia reconstruction using tissue expander was performed in three stages.

First stage was implantation of tissue expander, second stage involved framework fabrication using autogenous costal cartilage and implantation in the pocket and the third stage involved tragus and concha reconstruction. We evaluated 180 patients operated in our center during 2 years period. Patients were divided into two groups. Group 1 enrolled patients ranging from 4 to 9 years old (Total: 87 patients). Group 2 included patients ranging from 10 to 41 years old (Total: 93 patients).

**Fig. 1:**
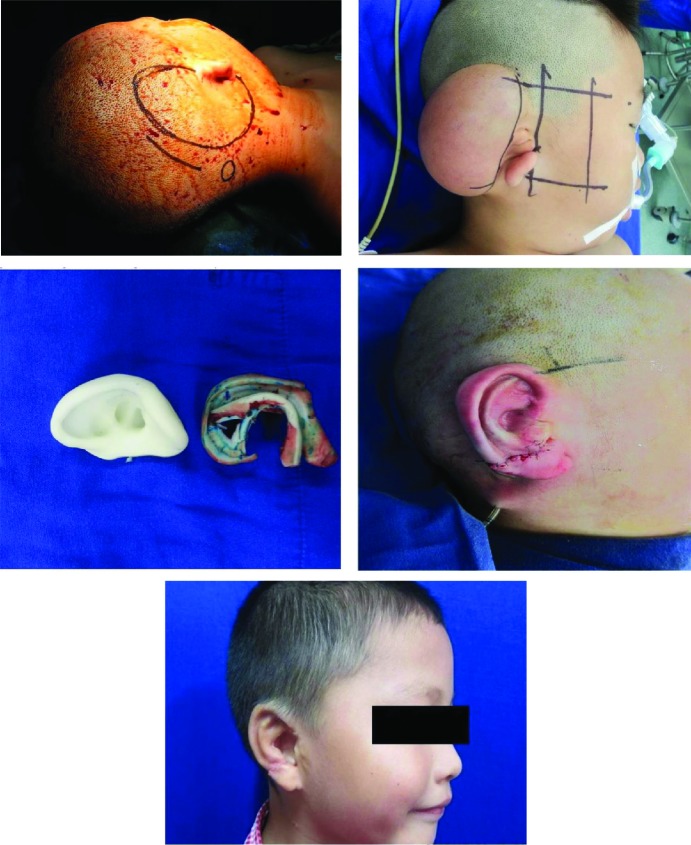
Process of ear reconstruction

Regarding stage I and implantation of tissue expander, the hair bearing part of the defective side was shaved before patient was shifted to the surgery room. Pre-surgical marking was done in the incision and remoted valve placement site as shown in Figure 1. In case of older patients, surgery was performed under local anesthesia whereas in younger patients, surgery was undertaken under general anesthesia. Approximately 3-4 cm incision was made at the temporal hairline and parallel to the tissue expander. The skin flap was elevated at subcutaneous level using scalpel and scissor. 

Hemostasis was maintained after careful observation. Tissue expander was checked fur any leakage and inserted into subcutaneous pocket. Totally, 80 mL or 100 mL kidney shaped silicon tissue expander was used based on size of the ear. Remote valve was placed underneath the skin in hair bearing area and below the incision site. Negative pressure drainage was applied and skin was closed in two layers. Suture was removed around 10 days after the surgery and first inflation with normal saline was done on the day patient comes to take the suture out. Then, for next 3 months inflation with normal saline was conducted on weekly basis. The second stage surgery was done one month after completion of the inflation.

Considering stage II and rib cartilage harvesting and ear reconstruction, in most cases, stage II surgery was performed after 4-5 months of implantation of tissue expander. After general anesthesia, incision site was marked on the ipsilateral side of the defect for harvesting costal cartilage. The measurement and marking for the positioning of the reconstructed ear was done based on contralateral normal ear as shown in Figure 1. In most cases, we harvested sixth, seventh and eighth costal cartilage. 

If the cartilage was not enough for the framework fabrication, we further harvested fifth or ninth costal cartilage. While harvesting cartilage, we left most of the perichondrium in situ to minimize chest wall deformity. In order to give three dimensional contour to the ear, we fabricated the framework in three different levels with different elevation. We used 3D printed ear model of the patient’s normal ear for the convenience of fabrication. At first main body of the framework was fabricated using seventh rib. The scapha and triangular fossa was carved, whereas the antihelix, superior and inferior crus of antihelix were formed by the other part of the cartilage. 

The eighth rib which was the thinnest one was used to construct the helix and crus helix of the framework. The third level formed the base of the framework, which maintained the prominence of the framework formed by the seventh rib. In case of adult who had larger cartilage, sixth and seventh cartilage for the entire fabrication was enough. All the structures were assembled using stainless steel wire and prolene to form a three-dimensional framework as shown in Figure 1. The extra piece of cartilage which was not used for the framework fabrication was buried beneath the skin in chest, which will be used for the framework elevation during stage 3 surgery.

The transposition of the earlobe was done to the desired level. The tissue expander was removed from its position and the portion of capsule on the undersurface of the skin flap was removed to obtain the better extensibility. Hemostasis was maintained and framework inserted into the pocket. After that, negative pressure suction catheter was inserted between framework and pocket and secured. The negative pressure suction catheter was generally removed on 5^th^ to 6^th^ operative day. Suture was removed on 12^th^ operative day and patient was generally discharged after the suture removal.

Regarding stage III and the framework elevation and tragus reconstruction, we generally performed stage three, six months after the stage two surgery based on the patient’s/family’s request. Some patients were satisfied with earlier surgery, whereas other patients needed framework elevation and tragus reconstruction. Surgery was performed under local anesthesia in adult patients and under general anesthesia in young patients. For the framework elevation, first the buried cartilage was removed from the chest and carved as required. After that, temporoparietal fascia flap was elevated and tunneled subcutaneously to cover posterior cartilage graft. 

After advancement of the retroauricular skin, the remaining defect was covered with the skin graft and secured with the bolster. Using the modified Kirkham method, transverse flap from the conchal area was doubled on itself, for the tragus formation.^8^ In some cases on patient’s/family’s request, in order to create pseudomeatus the vestigial cartilage remnant was excised to deepen the conchal floor, so that the conchal cavity was deepened down to the mastoid periosteum. This cavity was covered with the full thickness skin graft and secured with bolster. Correction of hypertrophic scar was done, whenever the case demanded.

## RESULTS

Our study included 180 patients who were operated for microtia using tissue expander and autogenous costal cartilage between 2013 and 2014. We operated patients ranging from 4 years to 41 years old. Out of 180 patients, 127 were male and 53 were female, and the male to female ratio was 2.4:1. In term of laterality, right side microtia accounted for 115 cases, left side account for 63 cases and 2 were bilateral cases of microtia. We divided total cases in our study into 2 groups to compare the surgical time, complications, tissue expansion and number of autogenous costal cartilage harvested (Table 1). 

**Table 1:** Comparison of the complications between two groups

**Complication**	**No. of cases in group one (4-9 years old)**	**No. of cases in group two (10-41 years old)**	**Total**	**Management**
1. Hematoma after stage I of surgery (early)	2	7	9	Surgical evacuation of hematoma
2. Surgical site infection and tissue expander exposure after stage I of surgery (late)	2	0	2	Removal of tissue expander, dressing and antibiotic
3. Infection after stage II of surgery (early or delayed)	8	9	17	Dressing, irrigation and antibiotic
4. Infection along with cartilage exposure after stage II of surgery (delayed)	6	2	8	Surgical debridement, excision of the exposed cartilage and/or closed/covered with local flap
5. Delayed hematoma after stage II of surgery	1	0	1	Surgical evacuation and closed with negative pressure drainage on site.
6. Wound dehiscence after stage II of surgery	1	0	1	Closed under local anesthesia
7. Severe infection followed by chondritis after stage II of surgery (delayed)	0	1	1	Multiple treatments which failed, ultimately framework removal
8. Infection and/or cartilage exposure after stage III of surgery (early and delayed)	4	3	7	Dressing, antibiotic, surgical debridement, covered with flap or primary closure
Total (%)	24 (27%)	22 (24%)	46 (25%)	

The first group included the patients between 4 and 9 years and second group included the patients between 10 and 41 years. There were no cases of perioperative complications in our study (Table 1). The first group included 87 cases (48%) and the second group included 93 cases (52%) (Table 1). There was no major difference in complication rate between two groups. Twenty four patients in group one suffered some types of complications, whereas twenty two patients in group two suffered from some type of complications. There were major differences between two groups in term of cartilage required for the framework fabrication (Table 1). 

**Fig. 2:**
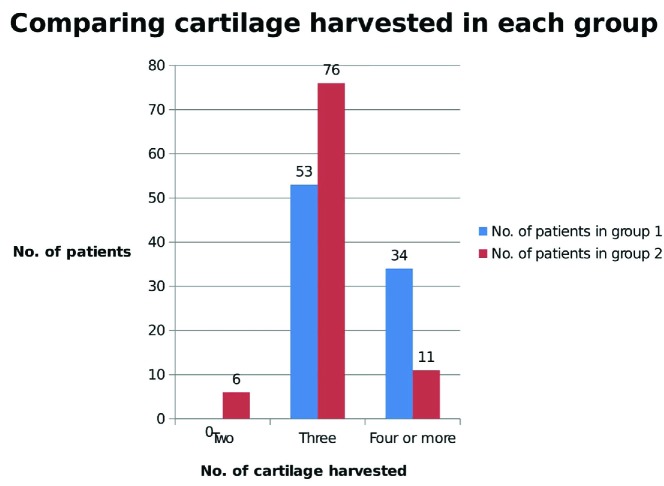
Comparing the number of costal cartilage harvested

Significantly larger number of patients in group one needed 4 or more cartilage tissues for the framework fabrication compared to patients in group two. The result was further illustrated in Figure 2. There was no significant difference between two groups in term of average surgical time. For the first group, the average time required to perform stage I and stage II surgery were 1 hour and 40 minutes and 4 hour and 54 minutes, respectively. Similarly, for group two, the average surgical time required to perform stage I and stage II surgery were 1 hour and 36 minutes and 4 hours and 48 minutes, respectively. Likewise, there was not also any significant difference in terms of tissue expansion. The average amount of fluid used for expansion was 136 mL in group one and 137 mL in group two.

## DISCUSSION

Reconstruction of microtia is one of the most challenging surgical procedure for the reconstructive surgeons because of its complex structure and need to construct near normal external ear. Over the past, various reconstructive methods have been used for the microtia reconstruction. Till date, surgical technique developed by Brent and Nagata are still most widely performed procedure for the reconstruction of microtia.^5^^,^^6^ The use of tissue expander is still controversial, but we have been using this method of reconstruction for more than 10 years with well acceptable results.

There is no consensus among the reconstructive surgeon regarding the timing of microtia surgery. Various factors like patient psychological status because of the abnormal ear, social stigma faced by the family and development of the rib cartilage must be considered, while deciding the appropriate age for reconstruction. According to the study, more than 20% of patients with microtia are associated with some kind of psychological issue like depression, interpersonal sensitivity and aggression.^9^

In our institution, we performed the microtia reconstruction starting from age four. We did not perform radiological analysis of the costal cartilage development before the surgery. In our study, there was a significance difference between the two groups in terms of costal cartilage harvested for the framework fabrication. Totally, 39% of cases in group one needed 4 or more cartilage, whereas only 12% of cases in group two needed 4 or more cartilage tissues. Also 7% of cases just needed 2 cartilage tissues for framework fabrication in group two. 

Ohora *et al.* reported that chest wall deformity is significantly higher if the cartilage is harvested from the children less than 10 years compared to the older group.^7^ In our study, more cartilage tissues were harvested from the younger patients (first group) compared to the older ones (second group) that might lead to the higher rate of chest wall deformity among the younger group of patients. Studies should be performed comparing the relationship between number of costal cartilage harvested and chest wall deformity. 

We suggest that the proper evaluation of costal cartilage development should be performed through radiological analysis before the microtia reconstructive surgery using the autogenous costal cartilage. The standard protocol should be developed for the timing of surgery taking into consideration the age, weight, psychological issue and development of the costal cartilage of the patient, who is undergoing microtia reconstructive surgery. Based on our study, we suggest that microtia surgery using tissue expander should be performed after 9 years of age to reduce the chances of chest wall deformity caused due to more cartilage harvest.

The overall complication in recipient site reached 25% (46 cases) in our study, but there was no significant difference among two groups. Although the complication rate was relatively high, but most of the complications were treated successfully. The most common complication we observed after implantation of tissue expander was hematoma. There were totally 9 cases of hematoma, 2 cases from the first group and 7 cases from the second group. The chief complain of the patient was severe pain on operated site and in case of children, they were constantly crying with pain.

 The presence of hematoma had a toxic effect which lead to flap necrosis and infection. So all the suspected cases of hematoma were well evaluated and transferred to operating room for evacuation. In our study, only one case needed removal of tissue expander because of uncontrolled bleeding within 24 hours of surgery, rest of the cases were managed without much difficulty. Additionally, 2 cases in our study had a severe infection on operated site, 3 weeks after stage I surgery. Infection was not controlled by antibiotic and dressing which lead to the exposure of the tissue expander. 

Ultimately, the tissue expander was removed from these 2 patients. The most common complication after stage II surgery was infection, which was meticulously managed with daily dressing and antibiotic. Furthermore, the exposure of the cartilage was observed in total 15 cases after either stage II or stage III surgery. The most common site for cartilage exposure was upper portion of the helical rim. We believe that the sharp edges of the cartilage might cause constant pressure on the overlying flap which lead to tissue necrosis and cartilage exposure. 

In most of the cases, primary closure was done after surgical debridement and excision of the exposed cartilage. Whereas, some cases needed local temporal flap or skin graft for the coverage. Due to the excised exposed cartilage, some cases were left with slightly deformed but acceptable shape of the ear. In our study, we encountered one severe case of complication after stage II surgery. Thirteen years old patient came to hospital, 40 days after stage II surgery with infection and necrosis of the flap. 

First the patient was treated with surgical debridement, local flap and broad spectrum IV antibiotic, but 10 days after surgery, the patients were again admitted with severe infection of the flap. At this stage, we used vacuum assisted closure (VAC) in order to reduce the bacterial load of the site, increase the blood flow and reduce the edema.^10^^-^^12^ All our attempts failed to preserve the framework, when patients developed chondritis, which left with no option than removing the framework.

Most common complications in our study included hematoma after stage I surgery, infection after stage II surgery and cartilage exposure after stage II surgery. Hematoma after implantation of tissue expander happened due to various factors like incomplete hemostasis during surgery, excessive crying due to pain post surgery, etc. We suggest meticulous hemostasis before the placement of tissue expander and proper pain medication to comfort the child after surgery. Other complications can be reduced by proper dressing and prophylactic antibiotic use. 

We were further educating parents about the ways to reduce infection after stage II surgery. Our study had some limitations which included failure to access the complications related to the donor site. Although more costal cartilage tissues were harvested from the donor site in younger group, we cannot guarantee the higher complications in that group. The other limitation included failure to track the minor complications, where patients might have undergone treatment in the local hospital. The cases of minor cartilage absorption might not be reported to the hospital.

In conclusion, microtia reconstruction using tissue expander and autogenous costal cartilage is a standard method of ear reconstruction with good satisfaction rate for the surgeon and the patient. The biggest advantage of tissue expander included the availability of well vascularized and non-hair bearing flap. Although, the overall complication rate was high, but most of them were easily manageable. Patients in age group of 4-9 years required more costal cartilage for framework fabrication compared to patients in older age group, which might significantly affect the chest development. 

This is also one of the significant finding in our study. We believe that standard protocol should be developed regarding the timing of the surgery for microtia patients taking into consideration the age, weight, psychological issue and pre–operative radiological analysis of the costal cartilage development of the patient. We suggest further research should be done comparing the number of costal cartilage harvested and chest deformity among the patients.

## CONFLICT OF INTEREST

The authors declare no conflict of interest.
